# Vortioxetine treatment for neuropathic pain in major depressive disorder: a three-month prospective study

**DOI:** 10.3389/fneur.2024.1398417

**Published:** 2024-07-03

**Authors:** Sinan Eliaçık, Ayse Erdogan Kaya

**Affiliations:** ^1^Department of Neurology, Hitit University School of Medicine, Çorum, Türkiye; ^2^Department of Psychiatry, Hitit University School Of Medicine, Çorum, Türkiye

**Keywords:** neuropathic pain, major depressive disorder, vortioxetine, Beck Depression Inventory, Beck Anxiety Inventory, cognitive function, quality of life, pain measurement

## Abstract

**Introduction and objective:**

Several studies revealed the therapeutic potential of vortioxetine (Vo) for pain. In this context, we aimed to evaluate the efficacy of Vo as a safe and tolerable novel pharmacologic agent in treating neuropathic pain (NP) in patients with major depressive disorder (MDD).

**Materials and methods:**

The population of this cross-sectional prospective study consisted of all consecutive patients who were newly diagnosed with MDD by a neurology doctor at a psychiatric clinic and had NP for at least 6 months. All patients included in the sample were started on Vo treatment at 10 mg/day. They were assessed with Beck Depression Inventory (BDI), Beck Anxiety Inventory (BAI), Self-Reported Leeds Assessment of Neuropathic Symptoms and Signs (S-LANSS), Douleur Neuropathique 4 Questions (DN4), Montreal Cognitive Assessment (MoCA), and Neuropathic Pain Impact on Quality of Life (NePIQoL) at the beginning of treatment and during the follow visits conducted at the end of the first, second and third months of the treatment. During these follow-up visits, patients were also queried about any side effects of Vo.

**Results:**

The mean age of 50 patients included in the sample, 76% of whom were female, was 45.8 ± 11.2 years. There was a significant reduction in patients’ NP complaints based on DN4 and S-LANNS, the subscales of NePIQoL, and significant improvement in MoCA. There was a significant reduction in patients’ NP complaints based on DN4 and S-LANNS scores and a significant improvement in scores of the subscales of NePIQoL and MoCA.

**Conclusion:**

The study’s findings indicate that Vo, with its multiple mechanisms of action, can effectively treat NP independently of its mood-stabilizing effect. Future indication studies for Vo are needed to establish Vo’s efficacy in treating NP.

## Introduction

Chronic pain is a devastating clinical situation characterized by persistent or recurrent pain lasting more than 3 months (1). There are a variety of chronic pain conditions with different etiologies, including neuropathic, visceral, musculoskeletal, and cancer-related pain (2). Patients with chronic pain often simultaneously experience depression associated with the stressful state of chronic pain. Reduced pain threshold, increased pain perception, more pronounced functional limitations, and worse analgesic response are common denominators of patients with chronic pain complicated by depression (1).

Vortioxetine (Vo) is a novel antidepressant with a multimodal mechanism of action (1–3). Vo, the chemical formula of which is 1-[2-(2,4-dimethyl phenyl sulfanyl)-phenyl]-piperazine, is a bis-aryl sulfanyl amine compound. Vo, like many other antidepressants, inhibits the serotonin transporter and, at the same time, modulates the activity of 5-Histamine (5-HT) receptors (2). With its antidepressant and anxiolytic effects, Vo is frequently used in the treatment of major depressive disorder (MDD) (4). Vo’s multimodal mechanisms of action enable it also to be used as a sleep modulator. Vo can also be used as a painkiller medication by reducing hyperalgesia and increasing analgesia (1, 2).

Vo has a better safety profile than other antidepressants due to a lower incidence of side effects such as weight gain, sexual dysfunction, and cardiovascular side effects, as well as more consistent procognitive effects (3). These advantages benefit specific patient groups, such as those with higher comorbidities and cognitive disorders (1–3).

Neuropathic pain (NP) is a highly complex chronic condition with perceptual and emotional components, affecting 7 to 10% of the population and often accompanied by anxiety, depression, or sleep disorders (2, 5, 6). Therefore, treatment approaches that consider mood and sleep disorders have come to the fore in treating NP (7).

In light of this information, we aimed to evaluate the efficacy of Vo as a safe and tolerable novel pharmacologic agent in treating NP in patients with MDD.

## Materials and methods

### Study design

This study was designed as a cross-sectional, prospective study. The study protocol was approved by the Hitit University School of Medicine Ethics Committee (2023-139). The study was conducted in accordance with the Strengthening the Reporting of Observational Studies in Epidemiology (STROBE) guidelines for reporting observational studies^1^ and the ethical considerations outlined in the Declaration of Helsinki. Written informed consent was obtained beforehand from the patients included in the study.

### Population and sample

The study population consisted of all consecutive patients newly diagnosed with MDD by a neurology doctor at a psychiatric clinic who had an NP for at least 6 months. MDD diagnosis was based on the diagnostic criteria outlined in the Diagnostic and Statistical Manual for Mental Disorders Fifth Edition (DSM-5) (8). Patients with polyneuropathy, entrapment neuropathy, and infective metabolic diseases that could cause NP and a patient with dizziness and not responding to medical treatment were excluded from the study. In the end, the study sample consisted of 50 patients. All patients included in the sample were started on Vo treatment at 10 mg/day. They were assessed with Beck Depression Inventory (BDI), Beck Anxiety Inventory (BAI), Self-Reported Leeds Assessment of Neuropathic Symptoms and Signs (S-LANSS), Douleur Neuropathique 4 Questions (DN4), Montreal Cognitive Assessment (MoCA), and Neuropathic Pain Impact on Quality of Life (NePIQoL) at the beginning of treatment and during the follow visits conducted at the end of the first, second and third months of the treatment. During these follow-up visits, patients were also queried about any side effects of Vo.

### Statistical analysis

The collected data were statistically analyzed using SPSS 26.0 (Statistical Product and Service Solutions for Windows, Version 26.0, IBM Corp., Armonk, NY, US, 2019) software package. Descriptive statistics were expressed as mean ± standard deviation and percentage values. The change in measurements over time was analyzed using the repeated analysis of variance (ANOVA) test. Differences in measurements conducted at different times were analyzed using the independent *t*-test between two groups and the one-way ANOVA test between three or more groups. Pearson’s correlation test was used to analyze the relationships between the measurements.

The significance of the findings obtained from repeated measurements was determined by Tukey’s method for multiple comparisons. In cases where the assumptions of normal distribution and repeated measures ANOVA were not met, the Friedman test was used to analyze the changes over time. Multiple comparisons of measurements found to be significant in the Friedman test were performed using the Durbin-Conover test. Probability (*p*) statistics of ≤0.05 were deemed to indicate statistical significance.

## Results

The mean age of 50 patients included in the sample, 76% of whom were female, was 45.8 ± 11.2 years. Most patients were secondary school graduates (64%) and married (72%). The sociodemographic and clinical characteristics of the patients included in the sample are given in Table 1.

**Table 1:** Sociodemographic and clinical characteristics of the patients.

	Overall (*n* = 50)
Age (year)^†^	45.8 ± 11.2
Age groups^‡^	
<55 years	38 (76.0)
≥55 years	12 (24.0)
Gender^‡^	
Female	37 (74.0)
Male	13 (26.0)
Education level^‡^	
Secondary school	32 (64.0)
High school	10 (20.0)
Bachelor/master	8 (16.0)
Marital status^‡^	
Married	36 (72.0)
Single	7 (14.0)
Divorced	7 (14.0)
Hypertension^‡^	4 (8.0)

^†^Mean ± Standard Deviation, ^‡^*n* (%).

As side effects of Vo, nausea was observed in six patients, dizziness in two patients, headache in one patient, diarrhea in one patient, and constipation in one patient during the three-month follow-up period, most of which occurred in the first 2 weeks after starting the treatment.

**Figure 1:**
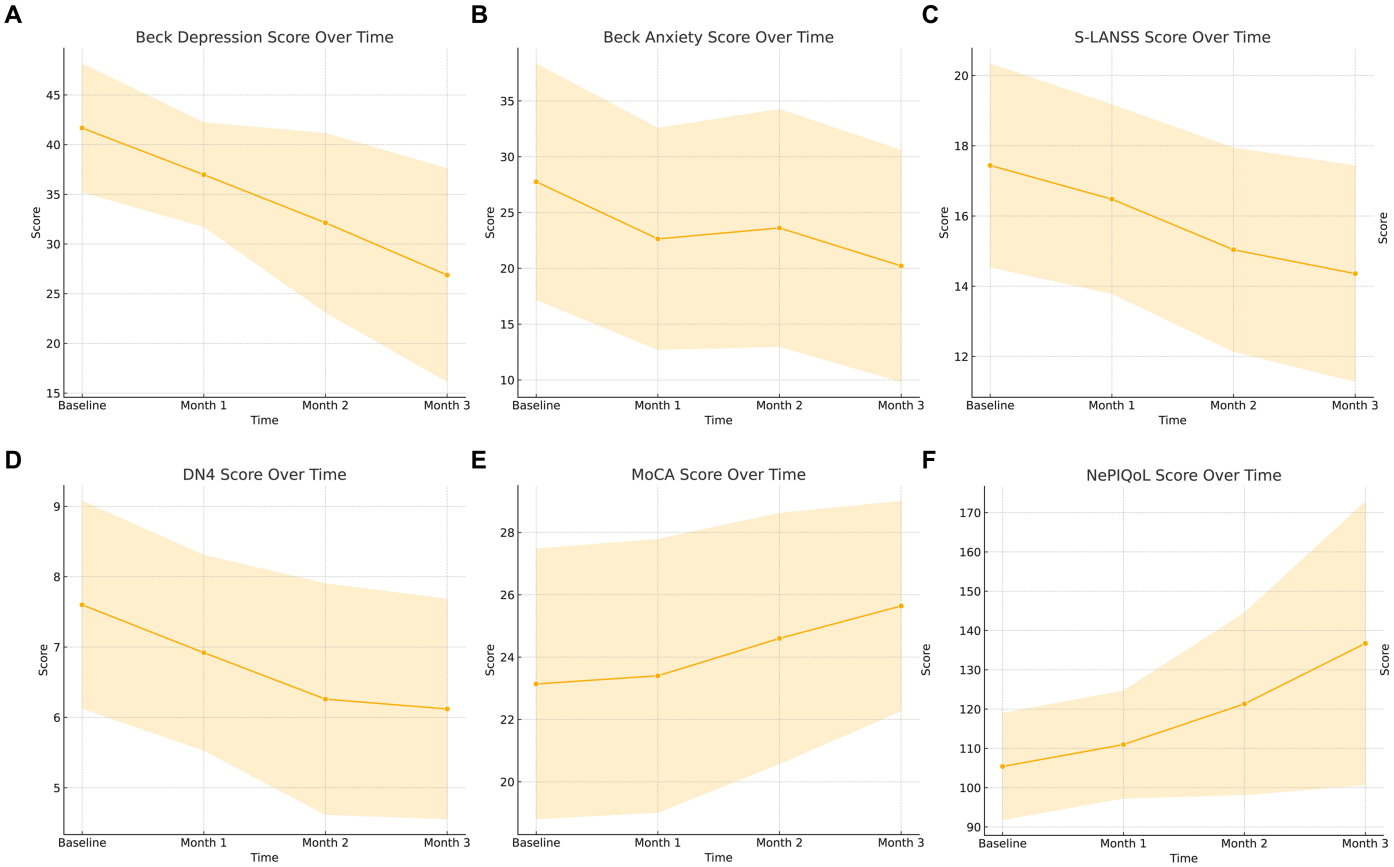
**(A–F)** Graphical summary of the questionnaires used in the study. MoCA, Montreal cognitive assessment test; S-LANSS, Self-Leeds Assessment of Neuropathic Symptoms and Signs; DN-4, Douleur Neuropathique 4 Questions; NePIQoL, Neuropathic pain quality of life questionnaire.

A significant gradual increase in mean MoCA score over the course of the follow-up period compared to baseline revealed that Vo had a significant positive effect on patients’ cognitive function (25.6 ± 3 vs. 23.1 ± 4.3, *p* < 0.001) (Table 2; Figure 1A).

**Table 2:** Changes in the assessment scores over the study period.

	Baseline	Month 1	Month 2	Month 3	*p*-value
MoCA score^†^	23.1 ± 4.3	23.4 ± 4.4	24.6 ± 4.0	25.6 ± 3.4	<0.001*
Beck depression Scale score^†^	41.7 ± 6.4	37.0 ± 5.2	32.1 ± 9.0	26.9 ± 10.7	<0.001*
Beck anxiety scale score^§^	26.0 [8.0–58.0]	20.0 [8.0–51.0]	23.0 [8.0–50.0]	18.0 [6.0–44.0]	<0.001**
S-LANSS score^§^	17.0 [13.0–24.0]	16.0 [13.0–23.0]	14.0 [10.0–23.0]	13.0 [9.0–23.0]	<0.001**
DN4 score^§^	7.5 [5.0–10.0]	7.0 [5.0–10.0]	6.0 [4.0–10.0]	6.0 [4.0–10.0]	<0.001**
NePIQoL score^†^	105.4 ± 13.5	111.0 ± 13.6	121.3 ± 23.1	136.7 ± 35.9	<0.001*
Symptoms^§^	20.0 [13.0–33.0]	21.0 [14.0–33.0]	24.0 [14.0–40.0]	30.0 [12.0–43.0]	<0.001**
Relationship^§^	13.0 [7.0–18.0]	13.5 [7.0–20.0]	15.0 [7.0–20.0]	16.0 [8.0–20.0]	<0.001**
Daily activities^†^	35.3 ± 7.7	36.5 ± 7.3	39.6 ± 9.5	44.0 ± 13.7	<0.001*
Psychological effects^§^	18.0 [8.0–27.0]	19.0 [8.0–29.0]	21.0 [8.0–36.0]	29.0 [8.0–40.0]	<0.001**
Personal care^†^	19.0 ± 4.8	19.6 ± 5.0	21.5 ± 5.6	23.2 ± 5.7	<0.001*

MoCA, Montreal cognitive assessment test; S-LANSS, Self-Leeds Assessment of Neuropathic Symptoms and Signs; DN-4, Douleur Neuropathique 4 Questions; NePIQoL, Neuropathic pain quality of life questionnaire. *Repeated Measures ANOVA. **Friedman Test. ^†^Mean ± Standard Deviation. ^§^Mean (minimum-maximum score). Bold to indicate values that are statistically significant.

The significant gradual decrease in mean BDI score over the course of the follow-up period compared to baseline revealed that Vo significantly reduced patients’ depression severity (26.9 ± 10.7 vs. 41.7 ± 6.4, *p* < 0.001, Figure 1B). Similarly, a significant decrease in median BAI score at the end of the follow-up period compared to baseline revealed that Vo significantly reduced patients’ anxiety severity (*p* < 0.001) (Table 2; Figure 1C). There was also a significant gradual decrease in the median S-LANNS score over the course of the follow-up period and a significant decrease in median DN4 at the end of the follow-up period compared to baseline (*p* < 0.001 for both cases) (Table 2; Figures 1D,E). The mean total NePIQoL score and mean NePIQoL subscale scores increased significantly over the course of the follow-up period compared to baseline (*p* < 0.001; Table 2; Figure 1F).

The changes in patients’ scores obtained from the assessment tools are shown in Figures 1A–F.

The mean BDI score of male patients was significantly higher than that of female patients (*p* = 0.042). There was no significant difference in other measurements between gender groups (*p* > 0.05; Table 3). There was also no significant difference in baseline mean BDI score between groups created based on marital status, i.e., married, single, and divorced (*p* = 0.057). On the other hand, there were significant differences between marital status groups in mean BDI scores assessed at the first-, second-, and third-month follow-up visits (*p* < 0.05; Table 3).

**Table 3:** Comparison of the beck depression scale scores obtained at different times in terms of gender, marital status, and educational status.

	Time/category	Women	Men		*p*-values
Beck depression scale	Baseline	36.11 ± 4.88	39.46 ± 5.56		0.042*
	Month 1	32.68 ± 5.80	34.54 ± 7.47		0.062*
	Month 2	31.84 ± 8.98	33.00 ± 9.29		0.692*
	Month 3	27.54 ± 11.46	25.00 ± 8.29		0.467*
	Time/category	Married	Single	Divorcee	*p*-values
	Baseline	40.58 ± 6.02	46.86 ± 5.96	42.14 ± 7.20	0.057**
	Month 1	35.97 ± 4.83	43.29 ± 4.57	35.86 ± 3.44	0.001**
	Month 2	30.50 ± 7.14	42.86 ± 10.78	29.86 ± 9.44	0.002**
	Month 3	24.31 ± 8.41	40.00 ± 10.58	27.00 ± 13.06	0.001**
	Time/category	Secondary school	High school	Bachelor/master	*p*-values
	Baseline	40.50 ± 5.53	41.30 ± 6.09	46.88 ± 8.29	0.039**
	Month 1	35.88 ± 4.58	36.70 ± 4.83	41.75 ± 6.02	0.014**
	Month 2	30.69 ± 8.63	33.80 ± 9.27	35.88 ± 9.73	0.283**
	Month 3	26.53 ± 10.85	28.30 ± 11.81	26.50 ± 9.89	0.900**

*Independent Samples T-Test. **One-Way ANOVA test. Bold to indicate values that are statistically significant.

There were significant differences in mean BDI scores assessed at baseline and first-month follow-up visits between groups created based on educational status, i.e., middle school graduates, high school graduates, and university graduates (*p* < 0.039 and *p* = 0.014). Patients with higher educational statuses had significantly higher baseline BDI scores assessed at baseline and first-month follow-up visits than those with lower educational statuses (Table 3).

The mean total NePIQoL score was significantly higher in patients over 55 than those younger than 55 (*p* = 0.009; Table 4).

**Table 4:** Comparison of the total NePIQoL scores according to the age groups.

Measurement	<55	≥55	*p*-value
Total NePIQoL score	131.21 ± 38.37	154.17 ± 19.14	0.009*

NePIQoL, Neuropathic pain quality of life questionnaire. Bold to indicate values that are statistically significant. *p < 0.05.

Analysis of the assessments conducted during the third-month follow-up visit revealed that the mean BDI score had a strong positive correlation with mean BAI score (*r* = 0.682) and a strong negative correlation with mean total NePIQoL score (*r* = −0.710; Table 5).

**Table 5:** Correlation analysis of the scores of the scales at the third month follow-up examination.

		Beck depression scale	Beck anxiety scale	MoCA	NePIQoL
Beck depression scale	*r*	1	0.682^**^	−0.223	−0.710^**^
	*p*	–	<0.001	0.120	<0.001
Beck anxiety scale	*r*		1	−0.142	−0.589^**^
	*p*		–	0.326	<0.001
MoCA	*r*			1	0.303^*^
	*p*			–	0.032
NePIQoL	*r*				1
	*p*				–

MoCA, Montreal cognitive assessment test; NePIQoL, Neuropathic pain quality of life questionnaire; ^*^p < 0.05; ^**^p < 0.01.

## Discussion

It is known that symptoms of depression and anxiety frequently occur in patients with chronic pain, as in patients with chronic diseases. Depression is the most common psychological complication and comorbid condition in patients with chronic pain. Depression accompanying chronic pain causes a decrease in pain threshold, an increase in nociceptive sensitivity, further functional limitations, and reduces the patient’s response to analgesia. There may also be a reciprocal relationship between chronic pain and anxiety and/or depression. It has been reported in the literature that pain and depression interact and that depression plays a role in the development and maintenance of chronic symptoms (9).

Although a wide variety of antidepressant medications are available for use, Vo differs from other antidepressant drugs in its combination of pharmacological properties (10). Vo’s mechanisms of action include increasing 5-HT levels by inhibiting the serotonin transporter. Thus, Vo offers an advantage compared to other antidepressants in that it has lower therapeutic dose ranges of 5–20 mg. In addition, Vo produces twice as much serotonin through 5HT receptors by different mechanisms, including antagonism of 5HTD1, 5HT3, and 5HT7, agonism of 5HT1A, and partial agonism of 5HT1B (11). Thus, Vo treatment is less likely to cause emotional blunting and is more efficacious in reducing anhedonia. Furthermore, various pharmacodynamic properties of Vo are more likely to be associated with higher antidepressive, antianxious, and procognitive effectiveness with potentially less weight gain (10). These advantages might be indicative of Vo in patients suffering from chronic pain.

We conducted our study evaluating the efficacy of Vo in patients with chronic NP, with reference to several studies in the literature in terms of Vo’s therapeutic potential for pain (12, 13). In addition to its agonist and antagonist effects on HT receptors, Vo exhibits antidepressant, procognitive, sleep-regulating, and anti-inflammatory activities through its impact on interleukin 4 (IL-4) and brain-derived neurotrophic factor (BDNF).

Vo is a novel pharmacologic agent with procognitive efficacy independent of mood improvement (14). Beyond its effect on serotonin transport, Vo accelerates the desensitization and disinhibition caused by the release of 5-HT. The antagonism of another receptor, 5-HT7, also plays a role in mood improvement by increasing serotonergic transmission (15, 16). It has been reported in the literature that Vo shows cognitive enhancing properties with improvements in different functions in humans (17–19). Decreased gabaergic transmission in the prefrontal cortex and hippocampus due to 5-HT3 receptor blockade, increased glutamatergic neurotransmission, and increased glutamate, acetylcholine, histamine, dopamine, and noradrenaline in the same regions with 5-HT1 receptor partial agonism were reportedly responsible for the positive effect of Vo in cognition. The antagonism of 5-HT7 increases acetylcholine and noradrenaline levels in the medial prefrontal region cortex. The enhancing effect on noradrenergic neurotransmission is also associated with the stimulation of 5-HT1A and the blockade of 5-HT3 receptors. These mechanisms of action of Vo support its efficacy in neuroplasticity (11, 20–22). Vo also affects the synaptic neuroplasticity of the brain by playing a role in neurogenesis by creating functional synapses, increasing BDNF levels and dendritic branching, and in dendritic spine maturation with mitochondrial support in the dentate gyrus of the hippocampus (23, 24). It has been reported that Vo induces the maturation of neurons by acting on dendrites (25). In our study, patients’ MoCA scores indicated cognitive improvement starting from the second follow-up visit. As a matter of fact, according to the results of a meta-analysis conducted in 2022, both 10 and 20 mg/day Vo doses positively affected cognitive symptoms in MDD patients (26).

Several hypotheses have been proposed in the literature regarding the basic mechanisms underlying the effect of Vo on pain. Accordingly, the blockade of serotonin transport by Vo, the increase of neurotransmitters such as noradrenaline and 5-HTin central and peripheral nervous system synapses, and its direct effect on receptor activity that can modulate pain transmission may explain the impact of Vo on pain (27, 28). In addition to its immunomodulatory, antioxidant, and anti-inflammatory effects, Vo is considered potentially effective in chronic pain by increasing BDNF levels (29, 30). Vo inhibits neuroinflammation and increases neurogenesis and neuroplasticity via 5-HT2b and 5-HT7 receptors (31).

The starting dose of Vo is 10 mg per day, but its 5 and 20 mg doses are also effective. The 20 mg dose of Vo has been associated with a more significant clinical response (32, 33). Many studies have found Vo is highly tolerable and more effective in MDD than many other antidepressant medications (34, 35). Vo also has a reducing effect on the anxiety levels that accompany MDD (36). It has been determined that the 8-week Vo treatment improved sleep quality and reduced sleep disorders. Vo has been shown to improve non-rapid eye movement (REM) sleep through its 5-HT receptor effects, increase slow-wave sleep, and improve sleep quality by suppressing REM sleep (37). Similarly, our patients’ NePIQoL scores indicated a statistically significant improvement in sleep-related problems with Vo treatment. The increase in NePIQoL scores as a result of the 10 mg/day Vo treatment we administered to all 50 patients for 3 months was significantly correlated with the decrease in their depression and anxiety-related complaints. In an experimental study, the effect of Vo on pain appeared after 7 days and gradually increased. Similarly, we observed a decrease in the pain complaints of many of our patients as of the end of the first month of treatment (28). Case series in the literature on Vo suggests that Vo may also have a positive effect on mood and restless legs syndrome symptoms through its activity on dopamine and gamma-aminobutyric acid (GABA) (38).

In today’s clinical practice, neurologists and physiotherapists widely use duloxetine, venlafaxine, and amitriptyline to treat various pain syndromes, including fibromyalgia and migraine (39). However, due to their side effect profiles, their use is limited, especially in elderly patients. Compared to these medications, Vo is a more effective and better-tolerated antidepressant at doses of 5–20 mg/day in individuals over 55 years of age, considering comorbid conditions. Similarly, our findings show that Vo can be used effectively and safely in patients over 55. Common side effects of Vo include nausea, headache, dizziness, and itching, while rare side effects include gastrointestinal disorders, insomnia, nasopharyngitis, dry mouth, urticaria, and suicidal ideation. No clinically significant impact of Vo treatment on vital signs, electrocardiogram values, liver enzymes, or body weight has been reported (40, 41). In our study, no patient discontinued medication or was excluded from the study due to side effects, except for one patient who was voluntarily excluded from the study due to persistent dizziness. The findings of this study support the clinical findings reported in the literature regarding the efficacy of Vo on NP (28, 42). In a study conducted on diabetic rats, Yucel et al. (43) showed that Vo, in addition to its neutral activity on glycemic control, significantly improved diabetes-induced hyperalgesia and allodynia responses without affecting motor coordination. Todorovic et al. (44) demonstrated the potential usefulness of Vo’s analgesic effect in treating inflammatory pain. Vo has been reported to be safe and tolerable at doses of 5 mg to 20 mg, and the most common side effects are nausea, headache, and diarrhea, in order of frequency (45).

Our study’s primary limitations are its small sample size, lack of a control group, lack of comparison with other antidepressants, and its single-center design. In our study, patients were not blinded to treatment and knew they were receiving Vo. Patients’ awareness that they were using Vo may have contributed to significant positive results obtained at 3-month follow-up due to positive expectations. Therefore, there is a need for future long-term, large-scale clinical studies featuring a double-blind design to reduce this potential bias. In addition, given its multimodal effect on serotonergic and other neurotransmitter systems, it is also necessary to consider Vo’s potential impact on other types of pain. Nevertheless, the beneficial effects of Vo on different pain conditions, such as osteoarthritis and phantom limb pain, have not yet been adequately studied. Therefore, future studies should also address the efficacy of Vo in various pain syndromes.

Depression is a multifactorial and clinical process with varying severity and symptoms. Chronic pain may inherently predispose individuals to psychiatric symptoms. Although these intertwined conditions are challenging to diagnose and treat, the doctor and the patient must overcome this process. Many animal, preclinical, and clinical studies have proven that some antidepressants show antinociceptive and analgesic effects and can be used in chronic pain. The goal of clinicians is to suppress many symptoms with a single tolerable medication for pain or MDD.

The mechanisms of action of each antidepressant or new active substance and their usefulness in relieving pain need to be investigated with both animal and human models. In this context, Vo emerges as a novel treatment option with proven tolerability and efficacy, attracting increasing attention. As with many molecules, clinical studies are needed to establish new indications for Vo, which started its journey as an antidepressant.

## Data availability statement

The datasets presented in this study can be found in online repositories. The names of the repository/repositories and accession number(s) can be found in the article/supplementary material.

## Ethics statement

The studies involving humans were approved by Hitit University Ethics Committee. The studies were conducted in accordance with the local legislation and institutional requirements. The participants provided their written informed consent to participate in this study. Written informed consent was obtained from the individual(s) for the publication of any potentially identifiable images or data included in this article.

## Author contributions

SE: Conceptualization, Data curation, Formal analysis, Funding acquisition, Investigation, Methodology, Project administration, Resources, Software, Supervision, Validation, Visualization, Writing – original draft, Writing – review & editing. AE: Funding acquisition, Methodology, Writing – original draft.

## Footnotes

^1^www.strobestatement.org
